# Fabrication of Multilayered Two-Dimensional Micelles and Fibers by Controlled Self-Assembly of Rod-Coil Block Copolymers

**DOI:** 10.3390/polym14194125

**Published:** 2022-10-02

**Authors:** Rui Qi, Wensheng Qi, Yin Zhang, Baohua Liu, Jian Wang, Hongmei Li, Haimei Yuan, Songzhi Xie

**Affiliations:** College of Food and Biological Engineering, Chengdu University, Chengdu 610106, China

**Keywords:** hierarchical micelles, rod-coil block polymer, multilayer, two-dimensional, fibers, π–π stacking

## Abstract

Fabricating hierarchical nanomaterials by self-assembly of rod-coil block copolymers attracts great interest. However, the key factors that affect the formation of the hierarchical nanomaterials have not been thoroughly researched. Herein, we have synthesized two diblock copolymers composed of poly(3-hexylthiophene) (P3HT) and polyethylene glycol (PEG). Through a heating, cooling, and aging process, a series of multilayered hierarchical micelles and fibers were prepared in alcoholic solutions. The transition from fibers to hierarchical micelles are strictly influenced by the strength of the π-π stacking interaction, the PEG chain length, and solvent. In isopropanol, the P3HT_22_-*b*-PEG_43_ could self-assemble into hierarchical micelles composed of several two-dimensional (2D) laminar layers, driven by the π-π stacking interaction and van der Waals force. The P3HT_22_-*b*-PEG_43_ could not self-assemble into well-defined nanostructures in methanol and ethanol, but could self-assemble into fibers in isobutanol. However, the P3HT_22_-*b*-PEG_113_ with a longer corona block only self-assembled into fibers in four alcoholic solutions, due to the increase in dissolving capacity and steric hindrance. The sizes and the size distributions of the nanostructures both increased with the increase in polymer concentration and the decrease in solvent polarity. This study shows a method to fabricate the hierarchical micelles.

## 1. Introduction

Solution self-assembly of block copolymers is a low-cost and effective method to fabricate the defined nanomaterials. By means of adjusting the chemical constituents and the environmental conditions, the block copolymer can self-assemble into various nanostructures, such as spherical micelles [1,2,3], one-dimensional (1D) micelles [4,5,6,7,8], two-dimensional (2D) micelles [9,10,11], hierarchical nanostructures [12,13,14,15], etc. Recently, fabrication of nanomaterials with hierarchical structures by self-assembly of the block copolymer attracts great interest. Owing to the huge specific surface, hierarchical nanomaterials have great application in many fields, such as templates [16,17,18,19], catalysis [20,21,22,23], sensors [24,25,26,27], etc. To prepare these hierarchical nanomaterials, a variety of methods have been developed [28,29,30]. Recently, many studies have proven that incorporating additional strong interactions to the assembly systems could favor the fabrication of hierarchical nanomaterials. These strong interactions include hydrogen bonding (H-bonding) [31,32,33], ionic recognition [34], metal coordination [35,36,37,38], crystallinity [39,40,41], π-π stacking interaction [42,43], etc. For example, Manners et al. [44] prepared a series of hierarchical nanostructures by H-bonding interactions of a homopolymer hydroxyl-functionalized poly(methylvinylsiloxane) (H-bonding donor) and the fibers with a corona block poly(2-vinylpyridine) (H-bonding acceptor). Li et al. [45] synthesized a photo-responsive diblock copolymer poly(ethylene glycol)-*b*-poly(*o*-nitrobenzyl-*L*-glutamate). Upon UV irradiation, the block copolymer could self-assemble into complex micelles induced by the ionic cross-link of Fe^3+^ and carboxylic acid. Due to the strong π-π stacking interactions, the π-conjugated rod-coil block copolymers show excellent capacity in the fabrication of hierarchical nanomaterials [46,47]. For example, Zhang et al. [48] synthesized an H-shaped block copolymer with bichromophoric perylenediimide as the π-conjugated core. Via the π-π stacking interactions, the H-shaped block copolymer could self-assemble into multilayered rectangles and pyramid-shaped parallelograms in the mixed solvents of chloroform and hexane. However, control over the shapes and sizes of the hierarchical nanomaterials is still challenging. Studying the key factors that affect the transition from the intermediate nanostructures into hierarchical nanomaterials is essential, which would be beneficial to control the morphologies of the nanostructures.

As one of the π-conjugated polymers, poly(3-hexylthiophene) is also widely used to prepare nanomaterials because of its excellent optoelectronic properties [49,50,51,52]. In previous studies [53], we prepared a series of regular 2D rectangular micelles by hierarchical self-assembly of poly(3-hexylthiophene) (P3HT)-*b*-polyethylene glycol (PEG) in isopropanol (*i*-PrOH). We proved that the diblock copolymers first self-assembled into fibers and then the fibers were further reorganized to form 2D rectangular micelles, induced by the π-π stacking interaction and the van der Waals force. However, the key factors that affect the transition from the fibers to the 2D rectangular micelles were still obscure. These factors would be beneficial to tailor the sophisticated structures of the hierarchical micelles and the fibers. To find out these factors, herein, we studied the effect of the strength of π-π stacking interaction, the PEG chain length, and solvent on the transition from the fibers to the hierarchical micelles. To achieve this object, the P3HT_22_ chain, possessing an intermediate strength of π-π stacking interaction, was chosen as the core-forming block in this study. The intermediate strength of π-π stacking interaction would provide an opportunity for us to controllably prepare the hierarchical micelles and fibers. In *i*-PrOH, the transition from fibers to hierarchical micelles was greatly influenced by the PEG chain length. The P3HT_22_-*b*-PEG_43_ could self-assemble into multilayered 2D hierarchical micelles in *i*-PrOH. In contrast, the P3HT_22_-*b*-PEG_113_ could only self-assemble into fibers. As the polymer concentration increased, the sizes of the multilayered 2D hierarchical micelles and fibers all increased. Notably, the P3HT_22_-*b*-PEG_43_ could self-assemble into hierarchical micelles composed of vertically stacked 2D lamellar layers and special hierarchical micelles composed of a series of linearly arranged 2D laminar layers, successively, with the increase in polymer concentration in *i*-PrOH. In addition, the self-assembly of the two block copolymers was also influenced by the solvent polarity. The P3HT_22_-*b*-PEG_43_ could not self-assemble into well-defined nanostructures in methanol and ethanol, but could self-assemble into fibers in isobutanol. The P3HT_22_-*b*-PEG_113_ all self-assemble into fibers in four solutions. The length of the fibers increased with the decrease in solvent polarity. This study offers insight into controlling the shapes and sizes of nanostructures.

## 2. Results and Discussion

### 2.1. Synthesis and Characterization of the Block Copolymers

The P3HT_22_-*b*-PEG_43_ and P3HT_22_-*b*-PEG_113_ were synthesized according to our previous report [53], as shown in Scheme S1. The two block copolymers were characterized by proton nuclear magnetic resonance spectroscopy (NMR) and gel permeation chromatography (GPC). The chemical shifts of the block copolymers and the molar ratios of the constituents are shown in Figure S1 and Figure S2, which correspond to the theoretical values. The GPC experiments (Figure S3) show that the number-average weights (*M*_n_) of the two block copolymers are 7173 g·mol^−1^ and 9339 g·mol^−1^, respectively, which are larger than the *M*_n_ (3886 g·mol^−1^) of the P3HT_22_ block. The corresponding polydispersity index (PDI) values of the two block polymers are 1.07 and 1.05, respectively, which indicate the narrow distributions. The results of ^1^H NMR and GPC demonstrate the successful synthesis of the two block copolymers.

### 2.2. The Effect of the PEG Chain Length on the Self-Assembly of the Block Copolymer

**Figure 1:**
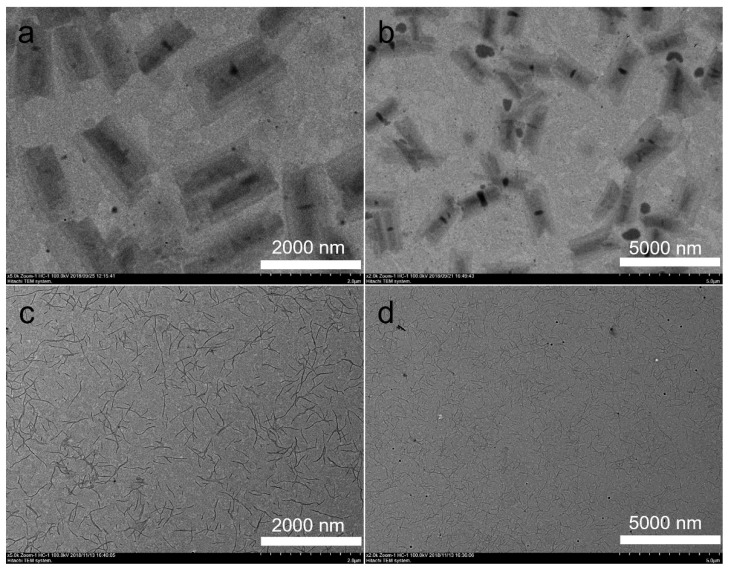
The TEM images of the nanostructures self-assembled by P3HT_22_-*b*-PEG_43_ (**a**,**b**) and P3HT_22_-*b*-PEG_113_ (**c**,**d**) in *i*-PrOH with the polymer concentration at ***c*** = 0.005 mg^.^mL^−1^.

**Figure 2:**
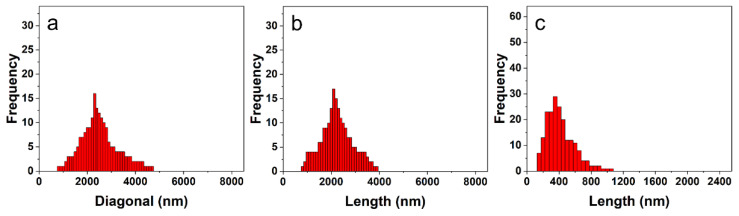
The size distributions of the nanostructures self-assembled by P3HT_22_-*b*-PEG_43_ (**a**,**b**) and P3HT_22_-*b*-PEG_113_ (**c**) in *i*-PrOH with the polymer concentration at ***c*** = 0.005 mg^.^mL^−1^. (**a**) The diagonal length distribution of the multilayered hierarchical micelles. (**b**) The length distribution of the 2D laminar layers included in multilayered hierarchical micelles. (**c**) The contour length of the fibers.

In previous studies [53], it was found that the sizes and aspect ratios of the 2D rectangular micelles could be controlled by varying the PEG chain length of the block copolymers with a constant long P3HT chain length. However, the shapes of the assemblies did not change with the variation of the PEG chain length, which did not offer adequate support to help us to understand the formation of the 2D micelles. Thus, in order to further understand the effect of the core-forming blocks and corona blocks on the formation of the 2D hierarchical micelles, the P3HT_22_ chain with an intermediate strength of π-π stacking interaction was selected as a constant core-forming block. In this study, the influence of the PEG chain length on the self-assembly of the block copolymers was first studied in *i*-PrOH at a constant polymer concentration (***c*** = 0.005 mg^.^mL^-1^). In solution in *i*-PrOH, the block copolymer P3HT_22_-*b*-PEG_43_ self-assembled into 2D hierarchical micelles, which was confirmed by transmission electron microscopy (TEM) (Figure 1a,b). It was found that these 2D hierarchical micelles were composed of several rectangular 2D laminar layers, which was different from the shape of the regular 2D rectangular micelles self-assembled by P3HT_22_-*b*-PEG_22_ reported in previous studies [53]. The number-average diagonal length (*D*_n_) of these multilayered hierarchical micelles was 2549 nm, which was slightly larger than the size (*D*_n_= 2388 nm) of the regular 2D rectangular micelles self-assembled by P3HT_22_-*b*-PEG_22_ [53]. This was probably owing to the imperfect stacking of the 2D laminar layers. Figure 2a shows that the diagonal length of these multilayered 2D hierarchical micelles ranged from 800 to 4700 nm, implying a wide distribution. This wide size distribution was also confirmed by the dispersity (*D*_w_/*D*_n_= 1.09, *D*_w_—the weight-average diagonal length) of these 2D hierarchical micelles. The size of the rectangular 2D laminar layers included in the multilayered 2D hierarchical micelles was also analyzed. The length of the 2D laminar layers ranged from 800 to 3900 nm (Figure 2b), indicating a wide distribution. The number-average length (*L*_n_) of the 2D laminar layers was 2359 nm, which was slightly larger than the length (*L*_n_= 2140 nm) of the regular 2D rectangular micelles self-assembled by P3HT_22_-*b*-PEG_22_ [53]. This may have contributed to the broad size distribution of the 2D laminar layers. The dispersity (*L*_w_/*L*_n_= 1.102, *L*_w_—the weight-average length) and the aspect ratio (*R*= 3.234) of the 2D laminar layers were both larger than the corresponding values of the regular 2D rectangular micelles self-assembled by P3HT_22_-*b*-PEG_22_ [53]. As the PEG chain length increased, the variation of the aspect ratio of the 2D nanostructures formed by P3HT_22_-*b*-PEG_m_ was in accordance with the corresponding change of the regular 2D rectangular micelles formed by P3HT_43_-*b*-PEG_m_ [53]. The standard deviation (*σ*) of the aspect ratio of the 2D laminar layers was 1.328, implying that the shape of the 2D laminar layers was not uniform. The wide size dispersity and the large standard deviation of the aspect ratio of the 2D laminar layers were probably due to the influences of the intermediate π-π stacking interaction and the variation in PEG chain length.

**Figure 3:**
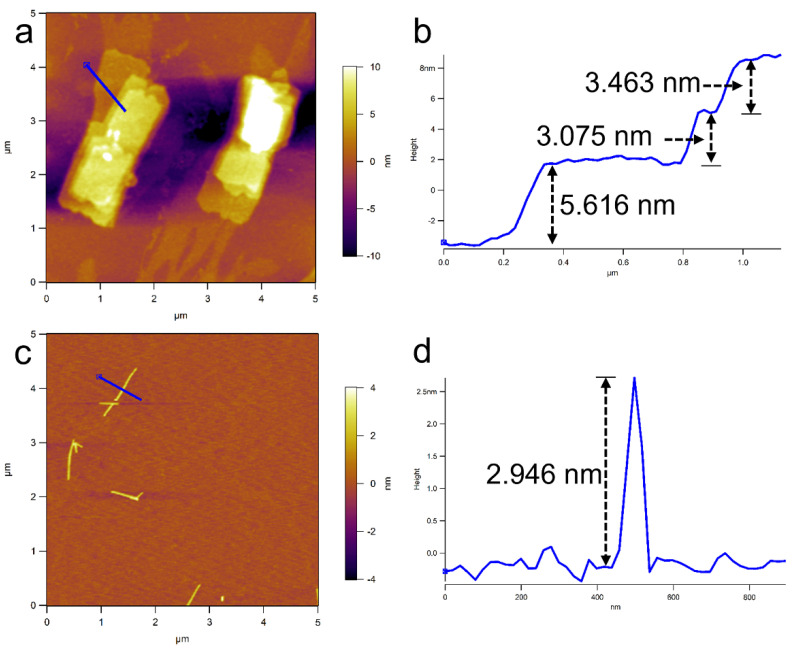
AFM images of the nanostructures self-assembled by P3HT_22_-*b*-PEG_43_ (**a**,**b**) and P3HT_22_-*b*-PEG_113_ (**c**,**d**) in *i*-PrOH with the polymer concentration at ***c*** = 0.005 mg^.^mL^−1^, the arrows represent the heights of each laminar layer or the single fiber.

**Figure 4:**
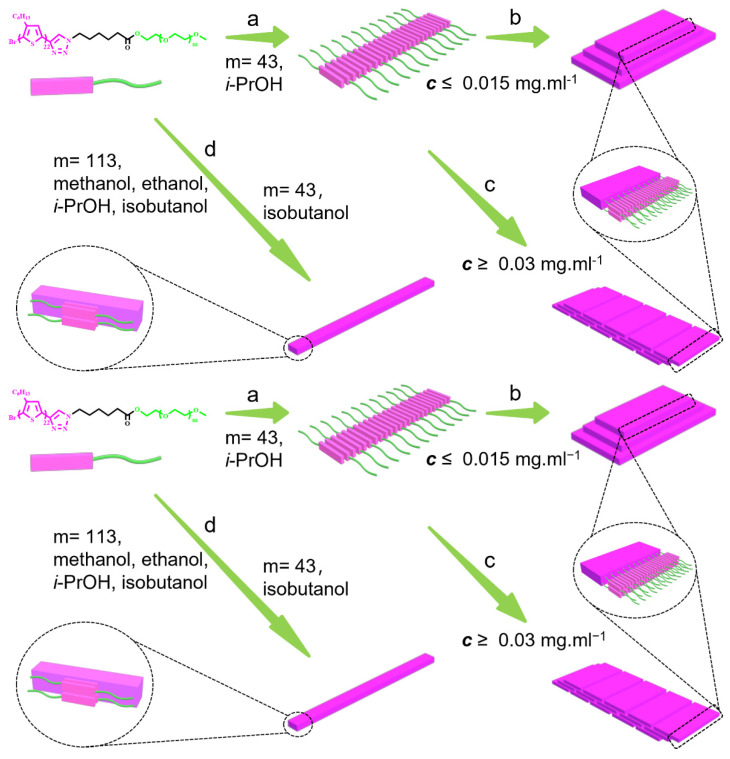
The probable formation processes of the nanostructures self-assembled by P3HT_22_-*b*-PEG_m_, (a) the transition fibers formed by P3HT_22_-*b*-PEG_43_ in *i*-PrOH during the cooling process, (b) the multilayered hierarchical micelles formed by P3HT_22_-*b*-PEG_43_ in *i*-PrOH when the polymer concentration is beblow 0.015 mg^.^mL^−1^, (c) the hierarchical micelles with linearly arranged 2D laminar layers formed by P3HT_22_-*b*-PEG_43_ in *i*-PrOH when the polymer concentration is above 0.03 mg.mL^−1^, (d) the fibers formed by P3HT_22_-*b*-PEG_43_ in isobutanol, or the fibers formed by P3HT_22_-*b*-PEG_113_ in methanol, ethanol, *i*-PrOH and isobutanol.

The multilayered structure of the 2D hierarchical micelles was also confirmed by atomic force microscopy (AFM) (Figure 3a). Figure 3b shows that the heights of the adjacent laminar layers were 3.463 nm, 3.075 nm and 5.616 nm, respectively. The difference in the heights of the adjacent layers indicates that the 2D laminar layers should be composed of a thinner nanostructure. To confirm the formation process of the multilayered 2D hierarchical micelles, we studied the morphologies of the assemblies formed by the P3HT_22_-*b*-PEG_43_ when the temperature of the solution decreased to 45 °C. Figure S4a shows the morphologies of the transition structures, which were composed of a series of linearly arranged fibers. This result demonstrates that the P3HT_22_-*b*-PEG_43_ first self-assembled into fibers, and then the fibers were further reorganized to form the multilayered 2D hierarchical micelles, as shown Figure 4a,b. The length of the 2D laminar layers should be equal to the contour length of the fibers. The formation process of the multilayered 2D hierarchical micelles is the same as that of the regular 2D rectangular micelles [53].

In the *i*-PrOH solution of the P3HT_22_-*b*-PEG_113_, there were no 2D micelles. The block copolymer all self-assembled into fibers (Figure 1c,d). The number-average contour length (*L*_n_) of these fibers was 440 nm, which was obviously smaller than the length of the 2D laminar layers included in the 2D hierarchical micelles formed by P3HT_22_-*b*-PEG_43_. Figure 2c shows that the contour length of these fibers ranged from 150 to 1050 nm, indicating a broad distribution. The dispersity (*L*_w_/*L*_n_= 1.172) of the fibers further confirmed the broad distribution. The AFM result (Figure 3c,d) shows that the height of the fibers was 2.946 nm. The width of the fibers was calculated to be 11.0 nm from the TEM and AFM results. According to the estimation of the sizes of the P3HT_22_ chain (as shown in Figure S5), the height of the fibers is slightly less than twice the length of side alkyl chain of P3HT, and the width of the fibers is slightly larger than the length of the P3HT_22_ chain. This result means that the fibers should be composed of two laminar layers of the P3HT_22_ chain, as shown in Figure S6.

The above results prove that the morphologies of the nanostructures were greatly influenced by the variation in PEG chain length, which should be due to the influences of the conjugated P3HT block and PEG block. It is known that the strength of π-π stacking interaction decreases with an decrease in P3HT chain length [54]. Meanwhile, the van der Waals interaction force of the block copolymer with a constant P3HT chain length decreases with an increase in PEG chain length. This is because the dissolving capacity increases with the increase in PEG chain length. In addition, the steric hindrance also increases with the increase in PEG chain length, which favors the formation of the fibers. Thus, the intermediate strength of the π-π stacking interaction and the weaker van der Waals interaction force cannot supply enough force to induce the fibers to form 2D micelles in the solution of the P3HT_22_-*b*-PEG_113_.

### 2.3. The Effect of the Polymer Concentration on the Self-Assembly of the Block Copolymer

**Figure 5:**
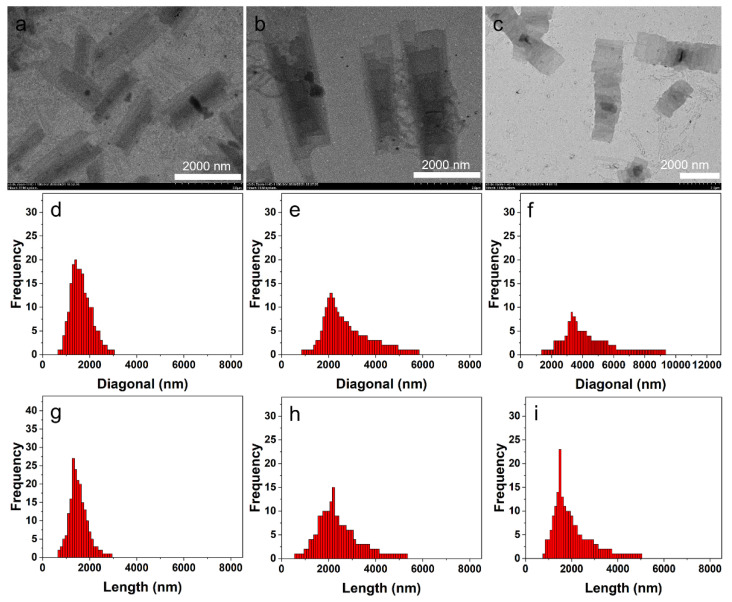
The TEM images and size distributions of the nanostructures self-assembled by P3HT_22_-*b*-PEG_43_ in *i*-PrOH with different polymer concentrations. TEM images of the multilayered hierarchical micelles in solutions at ***c*** = 0.001 mg^.^mL^−1^ (**a**), ***c*** = 0.015 mg^.^mL^−1^ (**b**), and ***c*** = 0.03 mg^.^mL^−1^ (**c**). The diagonal length distributions of the multilayered hierarchical micelles in solutions at ***c*** = 0.001 mg^.^mL^−1^ (**d**), ***c*** = 0.015 mg^.^mL^−1^ (**e**), and ***c*** = 0.03 mg^.^mL^−1^ (**f**). The length distributions of the 2D laminar layers included in multilayered hierarchical micelles in solutions at ***c*** = 0.001 mg^.^mL^−1^ (**g**), ***c*** = 0.015 mg^.^mL^−1^ (**h**), and ***c*** = 0.03 mg^.^mL^−1^ (**i**).

The influence of the polymer concentration on the self-assembly of the two block copolymers was also investigated. The self-assembly of P3HT_22_-*b*-PEG_43_ in *i*-PrOH at different polymer concentrations was studied first. In the solution at ***c*** = 0.001 mg^.^mL^−1^, the block copolymer still self-assembled into 2D hierarchical micelles composed of several 2D laminar layers (Figure 5a and Figure S7a). The *D*_n_ of the 2D hierarchical micelles and the *L*_n_ of the corresponding 2D laminar layers were 1699 nm and 1570 nm, respectively. The tiny difference in *D*_n_ of the 2D hierarchical micelles and in *L*_n_ of the corresponding 2D laminar layers indicates that the stacking of the 2D laminar layers was quite good, which could be further confirmed by the statistical size distributions. The statistical diagonal length of the 2D hierarchical micelles ranged from 700 to 3000 nm (Figure 5d). Meanwhile, the statistical length of the 2D laminar layers ranged from 700 to 2900 nm (Figure 5g). The polydispersity of the 2D hierarchical micelles (*D*_w_/*D*_n_= 1.074) and the 2D laminar layers (*L*_w_/**L**_n_= 1.068) implies good size distributions. The sizes of the 2D hierarchical micelles and their 2D laminar layers obtained in the solution (***c*** = 0.001 mg^.^mL^−1^) were obviously smaller than the corresponding sizes of the nanostructures obtained in the solution (***c*** = 0.005 mg^.^mL^−1^). As the polymer concentration increased from 0.005 to 0.015 mg^.^mL^−1^, the sizes of the 2D hierarchical micelles (Figure 5b and Figure S7b,c) and the corresponding 2D laminar layers obviously increased. The *D*_n_ of the 2D hierarchical micelles and the *L*_n_ of the 2D laminar layers were 2987 nm and 2598 nm, respectively. However, the size distributions of the corresponding nanostructures became broad. Figure 5e shows that the statistical diagonal length of the 2D hierarchical micelles ranged from 900 to 5800 nm. The statistical length of the 2D laminar layers ranged from 600 to 5300 nm, as shown in Figure 5h. The broad distributions were also confirmed by the polydispersity of the 2D hierarchical micelles (*D*_w_/*D*_n_= 1.173) and the 2D laminar layers (*L*_w_/*L*_n_= 1.190). When the polymer concentration increased from 0.015 to 0.03 mg^.^mL^−1^, amounts of special hierarchical micelles were found (Figure 5c and Figure S7d,e). It was easy to find that these special hierarchical micelles were composed of a series of linearly arranged 2D laminar layers. To further confirm the defined structures of these special hierarchical micelles, we studied the morphologies of the transition structures formed by P3HT_22_-*b*-PEG_43_ when the temperature of the solution (***c*** = 0.03 mg^.^mL^−1^) decreased to 45 °C. Figure S4b shows that the transition structures were composed of a series of linearly arranged fibers. These fibers arranged in a direction that was perpendicular to the length of the fibers. This result further proves that the special hierarchical micelles were formed by the reorganization of the fibers. The length of the linearly arranged 2D laminar layers should be equal to the length of the intermediate fibers. The probable formation process of these special hierarchical micelles is shown in Figure 4c. The *D*_n_ of the special hierarchical micelles was 4642 nm, which was observably larger than the *D*_n_ of the hierarchical micelles obtained in solutions with lower polymer concentration. This was due to the linear arrangement of the 2D laminar layers included in the special hierarchical micelles. The size distribution of the special hierarchical micelles was very broad, which could be confirmed by the dispersity (*D*_w_/*D*_n_= 1.211) and the statistical diagonal length. Figure 5f shows that the statistical diagonal length of the special hierarchical micelles ranged from 1400 to 9300 nm. The *L*_n_ of these linearly arranged 2D laminar layers was 2155 nm, which was slightly smaller than the *L*_n_ of the 2D laminar layers included in the hierarchical micelles obtained in the solution (***c*** = 0.015 mg^.^mL^−1^). This should contributed to the broad length distribution (*L*_w_/*L*_n_= 1.203) of the 2D laminar layers. Figure 5i shows that the statistical length of the 2D laminar layers ranged from 800 to 5100 nm, further implying a broad distribution. When the polymer concentration was above 0.05 mg^.^mL^−1^, the hierarchical micelles composed of a series of linearly arranged 2D laminar layers could still be found (Figure S7f). The sizes of these hierarchical micelles and the 2D laminar layers were very large and irregular, so they were difficult to measure. The increase in the sizes of the multilayered hierarchical micelles and the corresponding 2D laminar layers with the increase in polymer concentration is shown in Table 1. Table 1 shows that the aspect ratio (*R*) and the corresponding standard deviation (*σ*) of the 2D laminar layers increased with an increase in polymer concentration.

**Figure 6:**
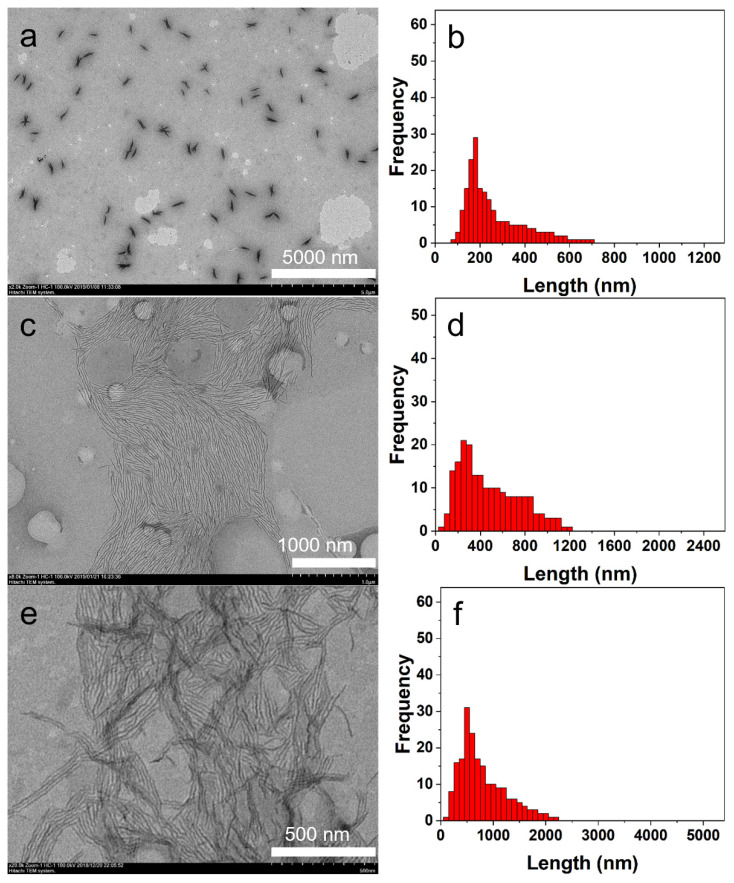
The TEM images and size distributions of the fibers self-assembled by P3HT_22_-*b*-PEG_113_ in *i*-PrOH with different polymer concentrations. The TEM images of the fibers in solutions at ***c*** = 0.001 mg^.^mL^−1^ (**a**), ***c*** = 0.015 mg^.^mL^−1^ (**c**), and ***c*** = 0.03 mg^.^mL^−1^ (**e**). The length distributions of the fibers in solutions at ***c***= 0.001 mg^.^mL^−1^ (**b**), ***c***= 0.015 mg^.^mL^−1^ (**d**), and ***c***= 0.03 mg^.^mL^−1^ (**f**).

In solution in *i*-PrOH, the shapes of the nanostructures formed by the P3HT_22_-*b*-PEG_113_ were not influenced by the variation in polymer concentration. The diblock copolymer P3HT_22_-*b*-PEG_113_ all self-assembled into fibers in a wide range of polymer concentrations (Figure 1c and Figure 6a,c,e). However, the sizes of the fibers were distinctly affected by varying the polymer concentration. Figure 6b shows that the contour length of the fibers formed in the solution (***c***= 0.001 mg^.^mL^−1^) ranged from 80 to 710 nm. In solution at ***c*** = 0.015 mg^.^mL^−1^, the contour length of the fibers ranged from 90 to1200 nm (Figure 6d), which was obviously larger than the lengths of the fibers obtained in the solutions (***c*** = 0.001 and 0.005 mg^.^mL^−1^). When the polymer concentration increased from 0.015 to 0.03 mg^.^mL^−1^, the contour length of the fibers continued to increase and ranged from 190 to 2200 nm (Figure 6f). Table 2 shows that the number-average contour length (*L*_n_) of the fibers increased with the increase in polymer concentration. The polydispersity (*L*_w_/*L*_n_) of all the fibers, ranging from 1.170 to 1.350, reveals that all the fibers have broad size distributions and that the size distribution of the fibers became broader with the increase in polymer concentration.

The variation in the sizes of the 2D laminar layers and fibers with the increase in polymer concentration could be explained as follows. As the polymer concentration increases, the Gibbs free energy of the system increases. The formation of nanostructures with larger sizes was more beneficial for reducing the Gibbs free energy of the system. Thus, the lengths of the 2D laminar layers and fibers both increase with an increase in polymer concentration. Meanwhile, the self-assembly of the block copolymers become more complicated in a higher polymer concentration, resulting in that the aspect ratio (*R*) and the corresponding standard deviation (*σ*) of the 2D laminar layers increased with the increase of the polymer concentration.

### 2.4. The Effect of the Solvent on the Self-Assembly of the Block Copolymer

The shapes and sizes of the nanostructures can usually be influenced by the solvent because the dissolving capacity, and the interactions of the conjugated blocks could be influenced by the solvent. Thus, the influence of solvents on the self-assembly of the two block copolymers in different solvents was also studied. Owing to their similar molecular structures, methanol, ethanol, and isobutanol were chosen as the additional selective solvents. In fact, the morphologies of the assemblies formed by the P3HT_22_-*b*-PEG_43_ were strictly influenced by the solvents. In methanol and ethanol, the diblock copolymer could not self-assemble into well-defined nanostructures (Figure S8a,b). Furthermore, a lot of red precipitates were found in the two solutions during the aging process. In solution in isobutanol (***c***= 0.015 mg^.^mL^−1^), the diblock copolymer could only self-assemble into the fibers (Figure S8c) with a number-average contour length (*L*_n_) of 1355 nm. The contour length of these fibers, ranging from 130 to 4000 nm (Figure S8d), implies that these fibers have a broad distribution, which was further confirmed by the dispersity (*L*_w_/*L*_n_= 1.572).

**Figure 7:**
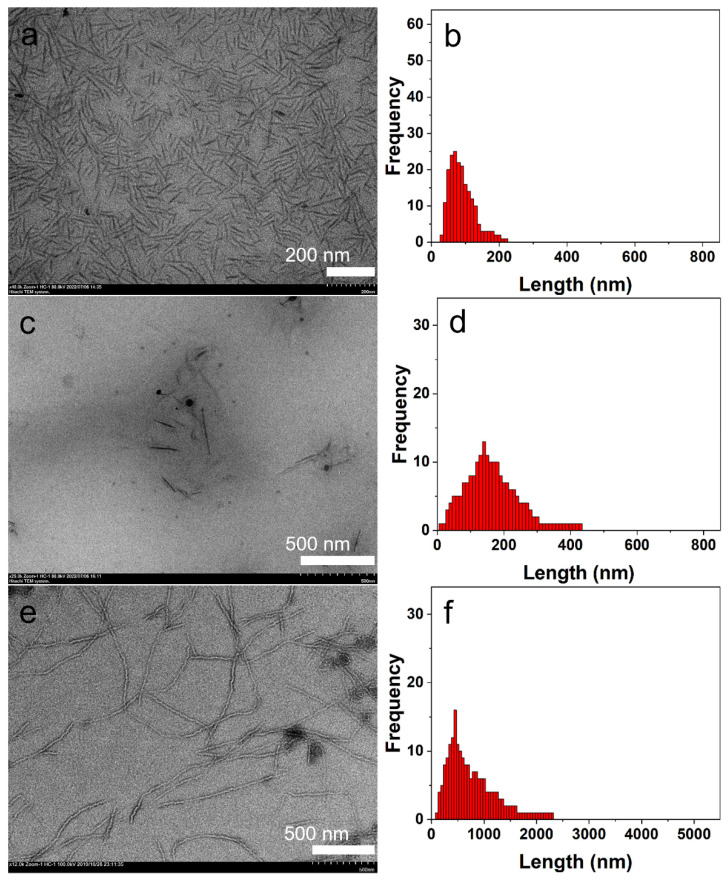
The TEM images and size distributions of the fibers self-assembled by P3HT_22_-*b*-PEG_113_ in different solvents at ***c*** = 0.015 mg^.^mL^−1^. The TEM images of the fibers in methanol (**a**), ethanol (**c**), and isobutanol (**e**). The length distributions of the fibers in methanol (**b**), ethanol (**d**), and isobutanol (**f**).

In contrast, P3HT_22_-*b*-PEG_113_ could be dispersed in all three alcohol solutions. The shapes of the nanostructures were not influenced by the variation of the solvent. In methanol, ethanol and isobutanol, the block copolymer all self-assembled into fibers (Figure 7a,c,e). However, the sizes of these fibers were greatly influenced by the variation of the solvent. In solution in methanol (***c***= 0.015 mg^.^mL^-1^), the block copolymer self-assembled into very short fibers. Figure 7b shows that contour length of the fibers obtained in methanol, ranging from 30 to 220 nm. In solution in ethanol (***c***= 0.015 mg^.^mL^−1^), the contour length of the fibers ranged from 20 to 430 nm (Figure 7d), which was obviously larger than the size of the fibers obtained in methanol. In solution in isobutanol (***c***= 0.015 mg^.^mL^−1^), the contour length of the fibers ranged from 140 to 2300 nm. The variation in *L*_n_ of these fibers formed in three solutions (shown in Table 2) further confirms the influence of the solvent on the sizes of the fibers. The size distribution (*L*_w_/*L*_n_) of these fibers also increased with a decrease in solvent polarity, as shown in Table 2.

The influence of the solvents on the self-assembly of the two block copolymers could be explained as follows. The dissolving capacity of the block copolymers P3HT_22_-*b*-PEG_m_ increased with the decrease in solvent polarity and the increase in PEG chain. In methanol and ethanol, the dissolving capacity of the P3HT_22_-*b*-PEG_43_ was too poor to self-assemble into well-defined nanostructures. In *i*-PrOH, the dissolving capacity of the P3HT_22_-*b*-PEG_43_ increased. The transition structures (fibers) could be reorganized to form multilayered hierarchical micelles, induced by the π-π stacking interaction and van der Waals force. In isobutanol, the dissolving capacity of the P3HT_22_-*b*-PEG_43_ greatly increased, leading to a decrease in the interactions of the P3HT_22_ block. There was no enough force to induce the fibers to form hierarchical micelles. Due to the long PEG chain, the dissolving capacity of the P3HT_22_-*b*-PEG_113_ in the four solvents was quite good. The P3HT_22_-*b*-PEG_113_ could only self-assemble into fibers in all four solvents, due to the increase in dissolving capacity and steric hindrance. The increase in the dissolving capacity of the P3HT_22_-*b*-PEG_m_ also leads to an increase in the dispersity of the fibers and a decrease in the length of the fibers.

## 3. Conclusions

In summary, we have prepared a series of multilayered hierarchical micelles self-assembled by P3HT_22_-*b*-PEG_43_ and fibers self-assembled by P3HT_22_-*b*-PEG_113_. The transition from the fibers to the hierarchical micelles has herein been proven to be influenced by the strength of the π-π stacking interaction, the PEG chain length, and solvent. The dissolving capacity and steric hindrance of the block copolymers both increase with an increase in PEG chain length. Meanwhile, the van der Waals force decreases with an increase in PEG chain length. In *i*-PrOH of the P3HT_22_-*b*-PEG_43_, the fibers (the transition structures) could be further reorganized to form multi-layered hierarchical micelles, driven by the intermediate strength of the π-π stacking interaction and the van der Waals force. In contrast, the P3HT_22_-*b*-PEG_113_ with a longer corona block could only self-assemble into fibers in *i*-PrOH, due to the decrease in van der Waals force and the increase in dissolving capacity and steric hindrance. In addition, the shapes and sizes of the nanostructures could be influenced by the polymer concentration and solvent. In *i*-PrOH, the P3HT_22_-*b*-PEG_43_ could self-assemble into the hierarchical micelles composed of vertically stacked 2D lamellar layers and the special hierarchical micelles composed of a series of linearly arranged 2D laminar layers, successively, with the increase of the polymer concentration. The P3HT_22_-*b*-PEG_43_ could not self-assemble into well-defined nanostructures in methanol and ethanol, but could self-assemble into fibers in isobutanol. The P3HT_22_-*b*-PEG_113_ could all self-assemble into fibers in methanol, ethanol, and isobutanol. The size and the size distribution of the fibers self-assembled by P3HT_22_-*b*-PEG_113_ both increase with an increase in polymer concentration and a decrease in solvent polarity. This study will offer insight into controlling the morphologies of nanostructures.

## Figures and Tables

**Table 1:** Summary data of the multilayered hierarchical micelles.

*c* (mg^.^mL^−1^)	*D*_w_ (nm)	*D*_n_ (nm)	*D*_w_/*D*_n_	*L*_w_ (nm)	*L*_n_ (nm)	*L*_w_/*L*_n_	*R*	*σ*
**0.001**	1823	1699	1.074	1677	1570	1.068	2.331	0.947
**0.005**	2790	2549	1.09	2600	2359	1.102	3.234	1.328
**0.015**	3503	2987	1.173	3091	2598	1.190	3.440	1.328
**0.03**	5622	4642	1.211	2593	2155	1.203		

*D*_w_ represents the weight-average diagonal length of the multilayered hierarchical micelles; *D*_n_ represents the number-average diagonal length of the multilayered hierarchical micelles; *L*_w_ represents the weight-average length of the 2D laminar layers included in multilayered hierarchical micelles; *L*_n_ represents the number-average length of the 2D laminar layers included in multilayered hierarchical micelles; *R* represents the aspect ratios of the 2D laminar layers; *σ* represents the standard deviations of the *R*.

**Table 2:** Summary data of the fibers.

Polymers	Solvent	*c* (mg^.^mL^−1^)	*L*_n_ (nm)	*L*_w_ (nm)	*L*_w_/*L*_n_
P3HT_22_-*b*-PEG_43_	isobutanol	0.015	1355	2131	1.572
P3HT_22_-*b*-PEG_113_	methanol	0.015	95	111	1.170
P3HT_22_-*b*-PEG_113_	ethanol	0.015	171	216	1.260
P3HT_22_-*b*-PEG_113_	isobutanol	0.015	845	1213	1.436
P3HT_22_-*b*-PEG_113_	*i*-PrOH	0.001	289	368	1.275
P3HT_22_-*b*-PEG_113_	*i*-PrOH	0.005	440	516	1.172
P3HT_22_-*b*-PEG_113_	*i*-PrOH	0.015	513	671	1.306
P3HT_22_-*b*-PEG_113_	*i*-PrOH	0.03	870	1173	1.348

*L*_w_ represents the weight-average contour length of the fibers, *L*_n_ represents the number-average contour length of the fibers.

## Data Availability

Not applicable.

## Supplementary Materials

The following supporting information can be downloaded at https://www.mdpi.com/article/10.3390/polym14194125/s1. Materials; Synthesis of the di-block copolymer; Preparation of polymer micelles; Polymer Characterization; The statistical sizes of the nanostructures; Scheme S1: Synthesis of P3HT_22_-*b*-PEG_m_; Figure S1: ^1^H-NMR spectrum of P3HT_22_-*b*-PEG_43_; Figure S2: ^1^H-NMR spectrum of P3HT_22_-*b*-PEG_113_; Figure S3: GPC trace (UV-vis) of P3HT_22_-*b*-PEG_43_ and P3HT_22_-*b*-PEG_113_ in THF; Figure S4: Images of the transition structures self-assembled by P3HT_22_-*b*-PEG_43_ when the temperature of the solutions decreased to 45 °C in *i*-PrOH at ***c*** = 0.005 mg^.^mL^−1^ (a) and ***c*** = 0.03 mg^.^mL^−1^ (b) during the cooling processes; Figure S5: The molecular conformation and the corresponding sizes of the P3HT_22_ chain, calculated with energy minimization (MM2) by ChemBio 3D Ultra; (a) width, (b) length; Figure S6: The probable structures of the fibers self-assembled by P3HT_22_-*b*-PEG_113_ in *i*-PrOH; Figure S7: The TEM images of the nanostructures self-assembled by P3HT_22_-*b*-PEG_43_ in *i*-PrOH with different polymer concentrations of ***c*** = 0.001 mg^.^mL^−1^ (a), ***c*** = 0.015 mg^.^mL^−1^ (b, c), ***c*** = 0.03 mg^.^mL^−1^ (d, e), and ***c*** = 0.05 mg^.^mL^−1^ (f); Figure S8: The TEM images and size distributions of the fibers self-assembled by P3HT_22_-*b*-PEG_43_ in different solvents at ***c*** = 0.015 mg^.^mL^−1^; the TEM images of the fibers in methanol (a), ethanol (b), isobutanol (c), and the length distributions of the fibers in isobutanol (d).
