# Supplementary material for: Fabrication of Multilayered Two-Dimensional Micelles and Fibers by Controlled Self-Assembly of Rod-Coil Block Copolymers
Source: Polymers (Basel). 2022 Oct 2;14(19):4125. doi: 10.3390/polym14194125 (PMC9571386; doi:10.3390/polym14194125)
Supplement: Supplementary file 1 [file polymers-14-04125-s001.zip › polymers-1916021-supplementary.pdf]

Supporting Information

Fabrication of multilayered two-dimensional micelles and fibers by controlled self-assembly of conjugated block copolymers

*Rui Qi, Wensheng Qi, Yin Zhang, Baohua Liu, Jian Wang, Hongmei Li, Haimei Yuan and Songzhi Xie**

College of Food and Biological Engineering, Chengdu University, Chengdu 610106, P. R. China

Content

1. Materials
2. Synthesis of the di-block copolymer
3. Preparation of polymer micelles
4. Polymer Characterization
5. The statistical sizes of the nanostructures
6. Additional Figures

1.Materials

The ethynyl-P3HT$_{22}$ chain, PEG$_{43}$-N$_3$ and PEG$_{113}$-N$_3$ were all obtained from the previous report [1]. Pentamethyldiethylenetriamine (PMDETA) and copper (I) bromide were purchased from Sigma-Aldrich and used without further purification. Tetrahydrofuran (THF) were purified before use with double alumina and alumina/copper catalyst drying columns from Anhydrous Engineering Inc. Chloroform (AR), methanol (HPLC), ethanol (HPLC), isopropanol (*i*-PrOH, HPLC) and isobutanol (*i*-BuOH, HPLC) were purchased from J&K Scientific Ltd. and used without further purification.

2. Synthesis of the di-block copolymer

The synthesis of the P3HT$_{22}$-*b*-PEG$_{43}$ and the P3HT$_{22}$-*b*-PEG$_{113}$ was shown in Scheme S1. Ethynyl-P3HT$_{22}$ (0.10 mmol), PEG$_m$-N$_3$ (0.30 mmol), and PMDETA (2.0 μL, 1.0 mmol) in freshly distilled dry THF (30 mL) were added to a one-neck round-bottom flask (100 mL) equipped with a magnetic stir bar and an argon-vacuum inlet/outlet. After three cycles of freeze-pump-thaw, the mixture was transferred into a glovebox under an argon atmosphere. CuBr (2.0 g, 1.0 mmol) was then added into the flask. The reaction mixture was stirred for 4 days at 40 °C. Cu/PMDETA was removed by passing the solution through an alumina column with THF as the eluent. The unreacted P3HT and unreacted PEG were removed by silica column chromatography using chloroform and chloroform/methanol (1:2) as the eluent, respectively. The desired product was obtained through column chromatography using chloroform/methanol (10:1) as the eluent. For P3HT$_{22}$-*b*-PEG$_{43}$ (254 mg, yield = 43.1%), GPC (Figure S3): M_n =7173 g·mol^{-1}, PDI = 1.07, and the molar ratio of thiophene group (HT) to ethylene glycol group (EG) was estimated to be 1:1.95 by the ^{1}H NMR spectrum (Figure S1). For P3HT$_{22}$-*b*-PEG$_{113}$ (311 mg, yield = 35.0%), G, GPC (Fig. S3): M_n =9339 g·mol^{-1}, PDI = 1.05, and the molar ratio of HT/EG was estimated to be 1:5.14 by the ^{1}H NMR spectrum (Figure S2). ^{1}H NMR (400 MHz, CDCl$_3$): δ (ppm) = 6.98 (s, 1H), 3.65 (m, 4H), 3.38 (s, 3H), 3.28 (t, 2H), 2.80 (t, 2H), 2.36 (t, 2H), 1.71 (t, 2H), 1.63 (m, 4H), 1.33-1.44 (m, 8H), and 0.91 (t, 3H).

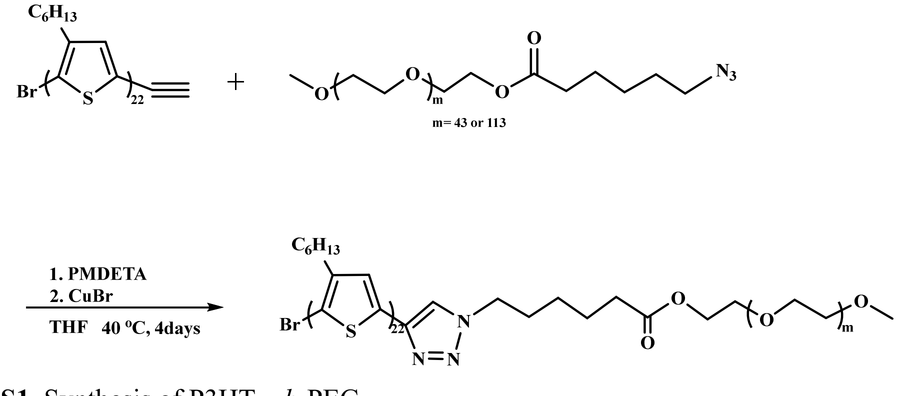

Scheme S1. Synthesis of P3HT$_{22}$-*b*-PEG$_m$

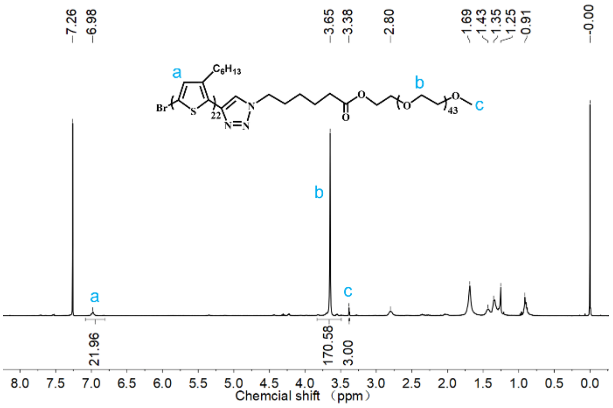

Figure S1. ^{1}H-NMR spectrum of P3HT$_{22}$-*b*-PEG$_{43}$.

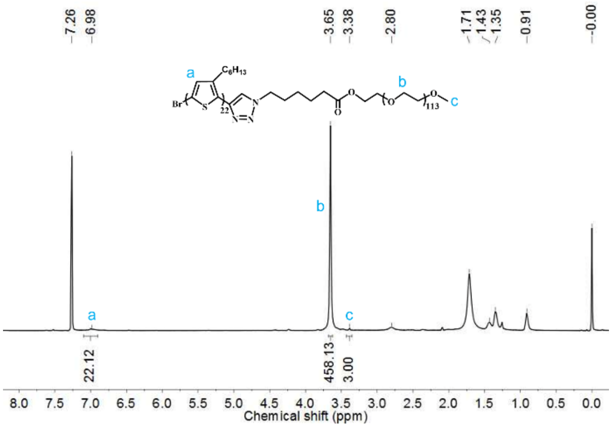

Figure S2. ^{1}H-NMR spectrum of P3HT$_{22}$-*b*-PEG$_{113}$.

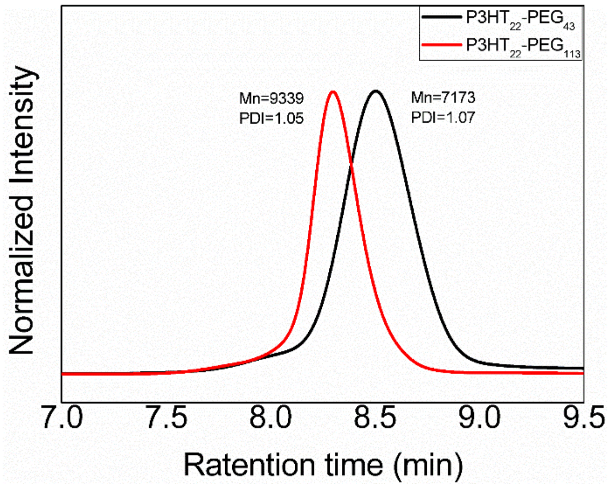

Figure S3. GPC trace (UV-vis) of P3HT$_{22}$-*b*-PEG$_{43}$ and P3HT$_{22}$-*b*-PEG$_{113}$ in THF.

3. Preparation of polymer micelles

The stoichiometric P3HT$_{22}$-*b*-PEG$_{m}$ in THF (0.5 mg·mL^{-1}) was first added to a 20 mL sealed vial. After solvent evaporation at rt (25 °C), 60 μL of THF was added to dissolve the polymer. Then, 6 mL of solvent was added dropwise to the vial with

magnetic stirring. After treatment with ultrasonic waves for 30 min, the solutions were heated at a fixed temperature (*i*-PrOH 80°C, *i*-BuOH 80°C, CH_3CH_2OH 75°C, CH_3OH 65°C) for 2 h and then slowly cooled down to rt (25 °C), followed by aging for 12 h. The obtained micelles were used for the respective experiments.

4. Polymer Characterization

^{1}H nuclear magnetic resonance (^{1}H NMR). The ^{1}H NMR spectra were measured on a Bruker AVANCE 400 MHz spectrometer with $CDCl_3$ as the solvent.

Gel permeation chromatography (GPC). GPC measurements were carried out on a Viscotek VE 2001 triple-detector gel permeation chromatograph equipped with an automatic sampler, a pump, an injector, an inline degasser, and a column oven at 40 °C. The elution columns consist of styrene/divinyl benzene gels with pore sizes of 500 and 100 000 Å. THF was used as the eluent, with a flow rate of 1.0 $mL \cdot min^{-1}$. Samples were dissolved in the eluent (1 $mg \cdot mL^{-1}$) and filtered with an organic-phase filter (polytetrafluoroethylene membrane with 0.45 mm pore size) before analysis. The calibration was conducted using a PolyCALTM polystyrene standard from Viscotek.

Transmission Electron Microscopy (TEM). The samples were prepared by drop casting 10 μl of the polymer solutions onto the carbon-coated copper grids, and then evaporating solvents. TEM images were obtained on a Hitachi HT7700 microscope operating at 100 kV and equipped with an AMF-5016 charge-coupled device camera.

Atomic Force Microscopy (AFM). The AFM samples were prepared by drop casting 10 μl of the polymer solutions onto the carbon-coated copper grids, and then evaporating solvents. Imaging was performed on an Asylum Research AFM in AC mode under ambient conditions. The silicon probe reflex coated with aluminum manufactured by Budget sensor Company was used as the sensor cantilevers. Images were obtained by IGOR Pro software (WaveMetrics Inc).

UV-vis Absorption Spectra (UV-vis). The UV-vis absorption spectra were recorded by a SHIMADZU UV3600 spectrophotometer.

5. The statistical sizes of the nanostructures

The sizes of each sample were obtained by analyzing two hundred micelles from TEM images using Digital Micrograph software (US Gatan company). The diagonal length was used to characterize the scale of the multilayered hierarchical micelles. The length was used to characterize the scale of the 2D laminar layers included in multilayered hierarchical micelles and the contour length of the fibers. The aspect ratio and the standard deviation were used to characterize the shape of the 2D laminar layers included in multilayered hierarchical micelles. The number-average diagonal length ($\boldsymbol{S}_n$) and the weight-average diagonal length ($\boldsymbol{S}_w$), the number-average length ($\boldsymbol{L}_n$), the weight-average length ($\boldsymbol{L}_w$), the aspect ratio ($\boldsymbol{R}$) and the standard deviation ($\boldsymbol{\sigma}$) of the assemblies were calculated by the following equations: (where $\boldsymbol{S}_i$, $\boldsymbol{L}_i$ and $\boldsymbol{R}_i$ are the sizes of individual micelle, respectively, $\boldsymbol{N}_i$ are the number of $\boldsymbol{S}_i$, $\boldsymbol{L}_i$ and $\boldsymbol{R}_i$).

$$\boldsymbol{S}_n = \frac{\sum_{i=1}^{N} N_i S_i}{\sum_{i=1}^{N} N_i}$$

$$\boldsymbol{S}_w = \frac{\sum_{i=1}^{N} N_i {S_i}^2}{\sum_{i=1}^{N} N_i S_i}$$

$$\boldsymbol{L}_n = \frac{\sum_{i=1}^{N} N_i L_i}{\sum_{i=1}^{N} N_i}$$

$$\boldsymbol{L}_w = \frac{\sum_{i=1}^{N} N_i {L_i}^2}{\sum_{i=1}^{N} N_i L_i}$$

$$\boldsymbol{R} = \frac{\sum_{i=1}^{N} N_i R_i}{\sum_{i=1}^{N} N_i}$$

$$\boldsymbol{\sigma} = \sqrt{\frac{\sum_{i=1}^{N} (R_i - \boldsymbol{R})^2}{\sum_{i=1}^{N} N_i}}$$

6. Additional Figures.

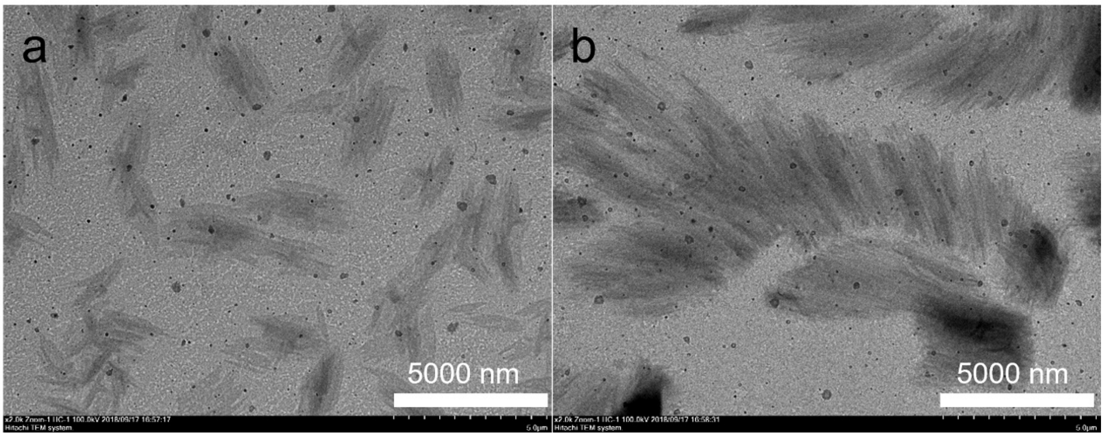

Figure S4. The images of the midterm states self-assembled by $P3HT_{22}$-*b*-PEG_{43} when the temperature of the solutions decreased to 45 ^{o}C in *i*-PrOH at ***c***= 0.005 $mg.mL^{-1}$ (a) and ***c***= 0.03 $mg.mL^{-1}$ (b), during the cooling processes.

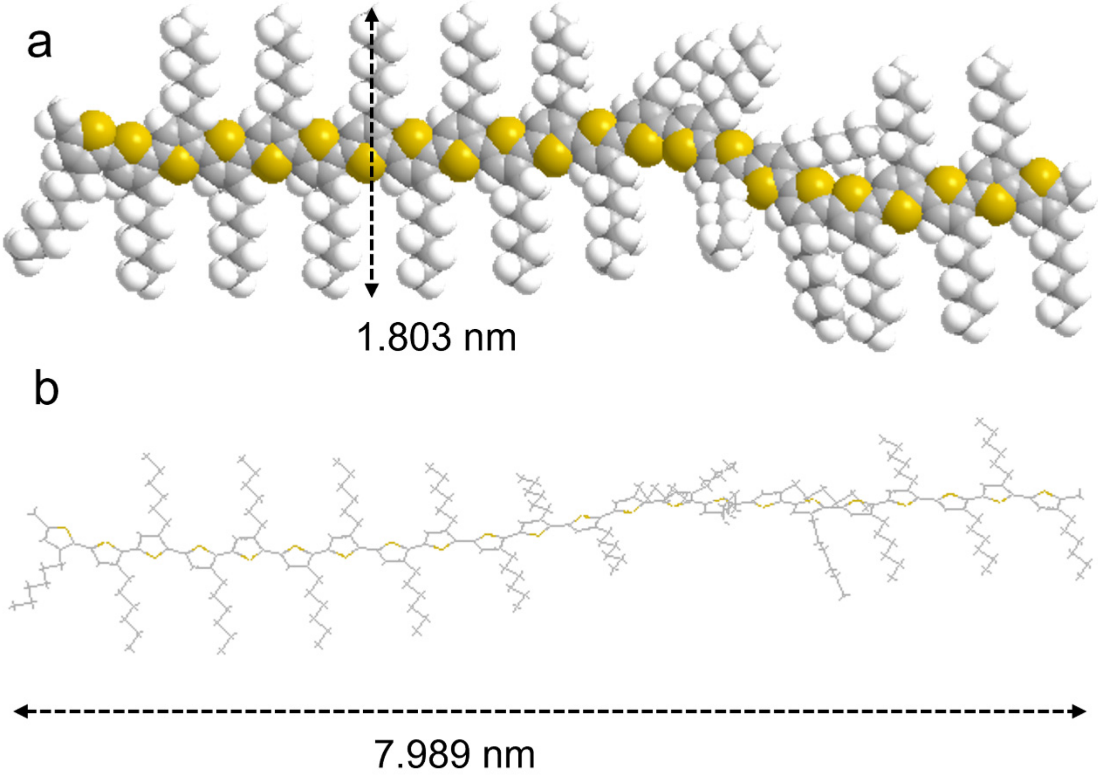

Figure S5. The molecular conformation and the corresponding sizes of the $P3HT_{22}$ chain, calculated with energy minimization (MM2) by ChemBio 3D Ultra, (a) width, (b) length.

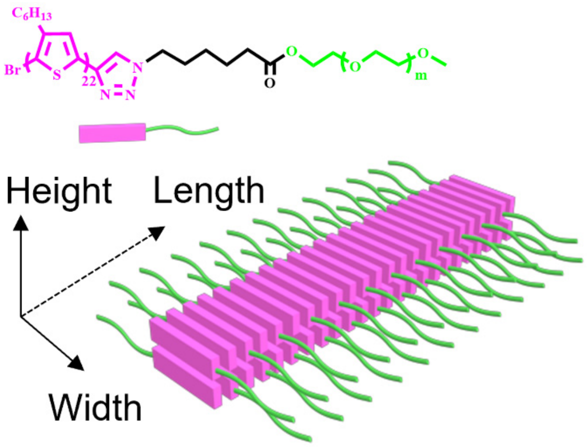

Figure S6. The probable structures of the fibers self-assembled by $P3HT_{22}$-*b*-PEG_{113} in *i*-PrOH.

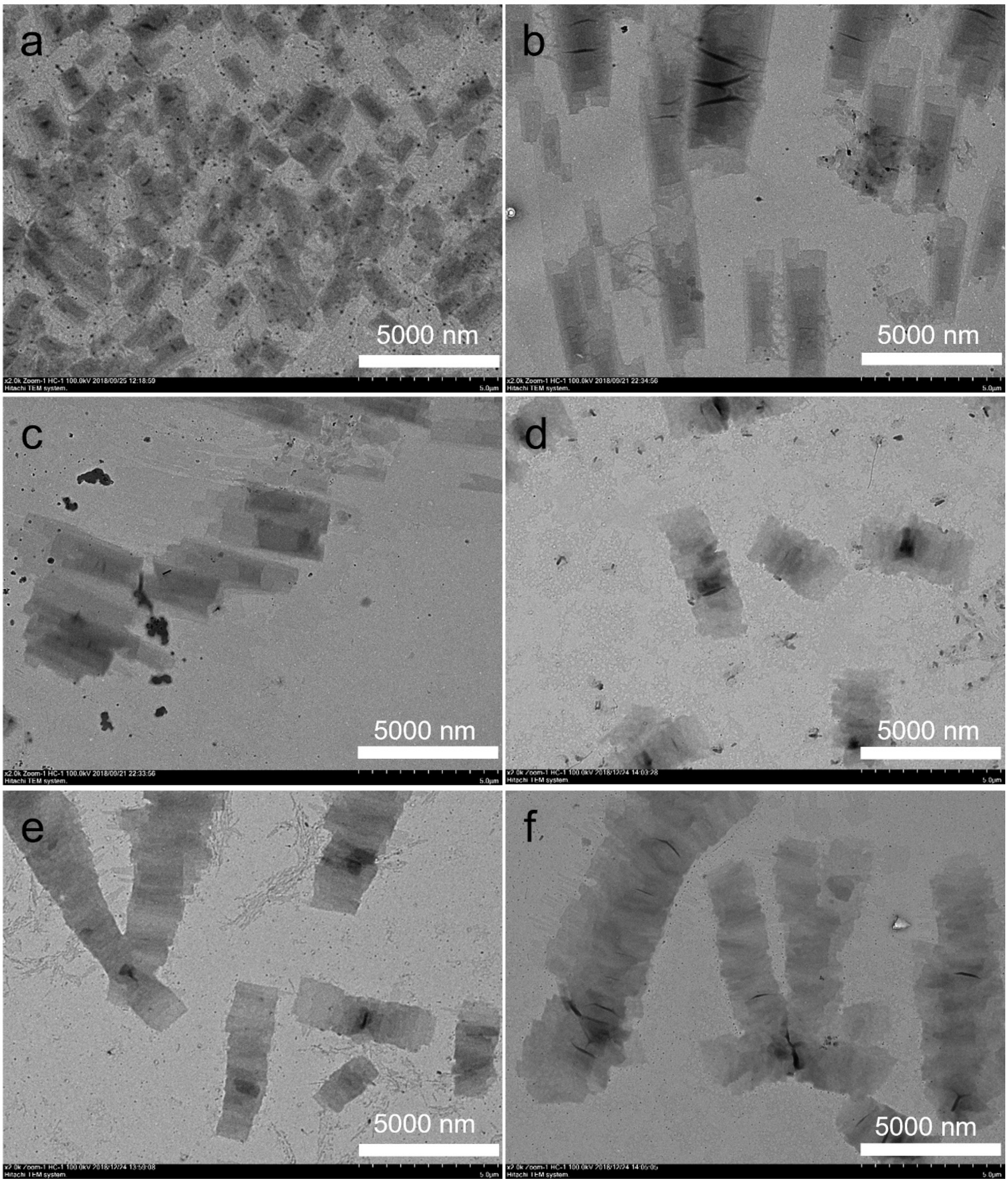

Figure S7. The TEM images of the nanostructures self-assembled by P3HT$_{22}$-*b*-PEG$_{43}$ in *i*-PrOH with different polymer concentrations, ***c***= 0.001 mg.mL^{-1} (a), ***c***= 0.015 mg.mL^{-1} (b, c), ***c***= 0.03 mg.mL^{-1} (d, e), ***c***= 0.05 mg.mL^{-1} (f).

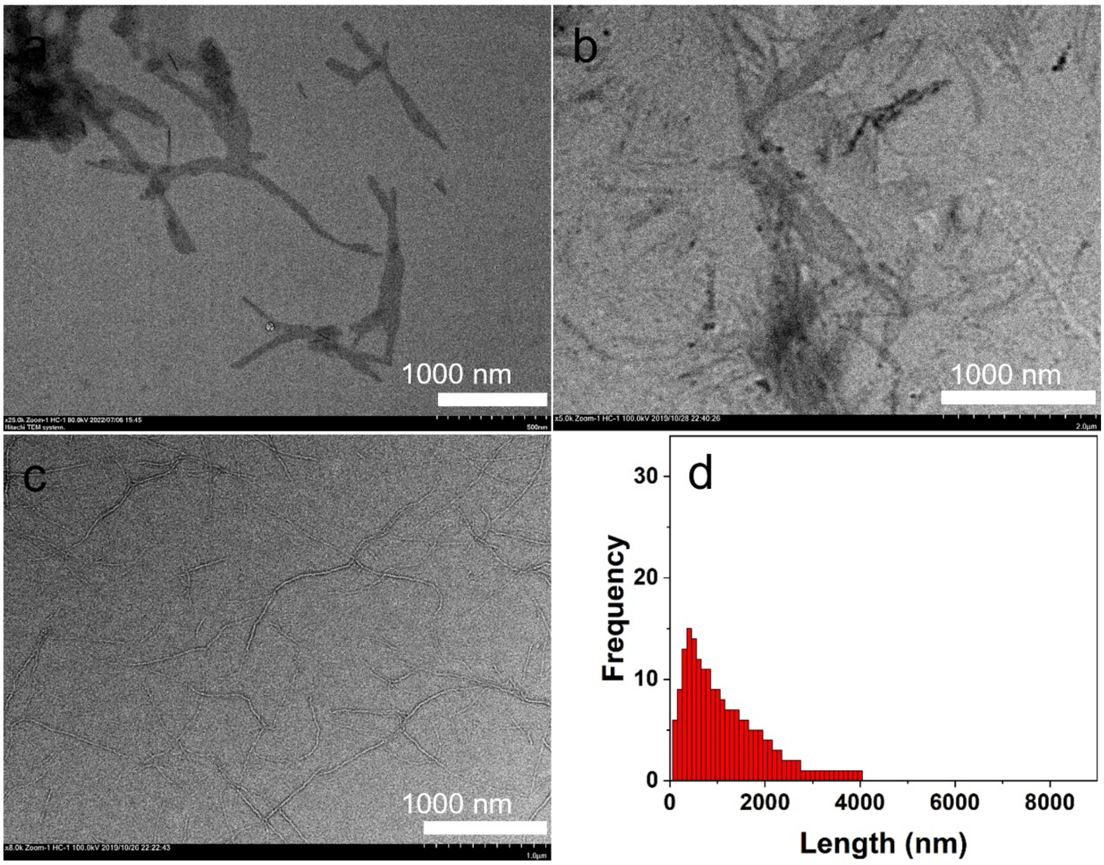

Figure S8. The TEM images and size distributions of the fibers self-assembled by P3HT$_{22}$-*b*-PEG$_{43}$ in different solvents at ***c***= 0.015 mg.mL^{-1}, the TEM images of the fibers in methanol (a), ethanol (b), isobutanol (c), the length distributions of the fibers in isobutanol (d).

References

1. Qi, R.; Zhu, Y. L.; Han, L.; Wang, M. J.; He, F., Rectangular Platelet Micelles with Controlled Aspect Ratio by Hierarchical Self-Assembly of Poly(3-hexylthiophene)-b-poly(ethylene glycol). *Macromolecules* **2020,** *53* (15), 6555-6565.
